# Artificial light at night can modify ecosystem functioning beyond the lit area

**DOI:** 10.1038/s41598-020-68667-y

**Published:** 2020-07-17

**Authors:** Simone Giavi, Sina Blösch, Guido Schuster, Eva Knop

**Affiliations:** 1 Institute of Ecology and Evolution, University of Bern, Baltzerstr. 6, 3012 Bern, Switzerland; 2 Bernese School of Agricultural, Forest and Food Sciences HAFL, Länggasse 85, 3052 Zollikofen, Switzerland; 3 Department of Electrical Engineering, University of Applied Sciences of Eastern Switzerland, Oberseestrasse 10, 8640 Rapperswil, Switzerland; 4 Agroscope, Agroecology and Environment, Reckenholzstrasse 191, 8046 Zürich, Switzerland; 5 Department of Evolutionary Biology and Environmental Studies, University of Zürich, Winterthurerstr. 190, 8057 Zürich, Switzerland

**Keywords:** Ecology, Behavioural ecology, Community ecology, Ecosystem ecology, Ecosystem services

## Abstract

Artificial light at night (ALAN) is a relatively new and rapidly increasing global change driver. While evidence on adverse effects of ALAN for biodiversity and ecosystem functioning is increasing, little is known on the spatial extent of its effects. We therefore tested whether ALAN can affect ecosystem functioning in areas adjacent to directly illuminated areas. We exposed two phytometer species to three different treatments of ALAN (sites directly illuminated, sites adjacent to directly illuminated sites, control sites without illumination), and we measured its effect on the reproductive output of both plant species. Furthermore, in one of the two plant species, we quantified pre-dispersal seed predation and the resulting relative reproductive output. Finally, under controlled condition in the laboratory, we assessed flower visitation and oviposition of the main seed predator in relation to light intensity. There was a trend for reduced reproductive output of one of the two plant species on directly illuminated sites, but not of the other. Compared to dark control sites, seed predation was significantly increased on dark sites adjacent to illuminated sites, which resulted in a significantly reduced relative reproductive output. Finally, in the laboratory, the main seed predator flew away from the light source to interact with its host plant in the darkest area available, which might explain the results found in the field. We conclude that ALAN can also affect ecosystem functioning in areas not directly illuminated, thereby having ecological consequences at a much larger scale than previously thought.

## Introduction

Artificial light at night (ALAN) is a relatively new global change driver, which is worldwide rapidly increasing^1^. It has various ecological consequences as it can cause alterations in physiology and behaviour of organisms, thereby increasing mortality, reducing reproduction as well as altering species abundances and community composition^1–5^. For example, many animals have been shown to be attracted^6,7^ or repelled^8–11^ by light. There is increasing evidence that ALAN has also consequences for species interactions: ALAN has been shown to alter mutualistic interactions, for example to disrupt nocturnal plant-pollinator interactions with negative consequences for the pollination service the nocturnal pollinators provide to the plants^12^. However, most of the research so far has focused on the effect of ALAN on predation.^8,11,13–16^.

These studies show that ALAN can reduce or increase species interactions and ecosystem functioning, and that the effect often depends on light quality and quantity^5,17^. However, so far, research has mostly focused on the impact ALAN has on species interactions and ecosystem functioning in directly illuminated areas^5,12,18,19^, ignoring what happens in areas in the surroundings of the illuminated area. Yet animals such as moths, might be attracted from long distances to the illuminated area potentially leading to lowered densities, interaction frequencies and ecosystem functioning in the dark surroundings^20,21^. Alternatively, they might be deterred by ALAN leading to an accumulation in the dark areas adjacent to the illuminated areas, with consequences for species interactions and ecosystem functioning^20,21^. Here we therefore experimentally tested whether the effect of ALAN on ecosystem functioning can also propagate beyond the directly illuminated area. In particular, we focused on the outcomes of mutualistic and antagonistic interactions, in particular the pollination service provided by insects and pre-dispersal seed predation, respectively. We hypothesized that (a) the reproductive output of a total of two model plant species is reduced on sites directly illuminated compared to dark control areas, and that (b) this effect propagates to the dark areas adjacent to areas directly illuminated by street lamps. Moreover, we hypothesized that (c) there are similar effects for pre-dispersal seed predation of one of our model plants. Finally, we hypothesized that (d) a reduction in pre-dispersal seed predation in areas adjacent to directly illuminated areas compensates for the negative effects of ALAN on pollination, resulting in a neutral effect on plant relative reproductive output.

**Figure 1:**
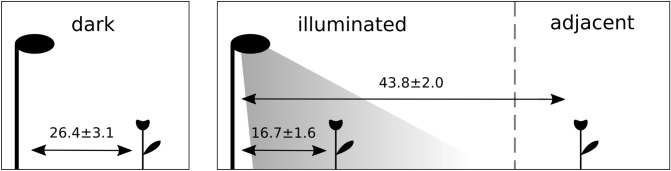
Schematic drawing of the field experiment set up and light treatments. Dark: plants exposed on dark control sites; illuminated: plants exposed on illuminated sites; adjacent: plants exposed to a dark site but adjacent to the illuminated site. Light intensity in dark and adjacent treatment is comparable, see text for details. Mean distance (in meters) ± s.e.m. of the phytometers from the lamp (illuminated and adjacent sites) and fake lamp (dark sites) are shown.

To test these hypotheses, we experimentally installed commercial LED street lamps on a total of four unmanaged meadows, which never experienced illumination before in a landscape with relatively low levels of light pollution. Another four unmanaged meadows were left untreated as control sites. On all these sites we exposed a total of 97 potted plants of two species, namely *Silene latifolia* (Caryophyllaceae) and *Epilobium angustifolium* (Onagraceae), to three different light treatments: direct illumination (further referred to illuminated sites), no direct illumination (i.e. dark locations) but adjacent to the illuminated sites (further referred to as adjacent sites), and no direct illumination and also no illumination in the vicinity (further referred to dark sites, Fig. 1). Both plant species were used to investigate the impact of the three light treatments on the reproductive output. Furthermore, one of the two plant species (*S. latifolia*) was used to investigate the effect of the light treatment on pre-dispersal seed predation: *S. latifolia* is involved in a specialized nursery pollination system, which means that the females of the moth species of the genus *Hadena sp*., often lay eggs into the female flowers when pollinating them^22^. The larvae then consumes the seeds of the pollinated fruits, often also infecting a second or third fruit^22^. As species of *Hadena sp*. are the main pollinator of *S. latifolia*, seed predation is relatively frequent.

In addition to the field experiment, we established an experiment in the laboratory with *S. latifolia* and *H. bicruris* in order to test for the impact of ALAN on the number of interactions of the moth with the flower and its egg laying behavior under controlled conditions, which most likely explains the results observed in the field experiment.

## Results

### Effect of ALAN on plant reproductive output and pre-dispersal seed predation

**Figure 2:**
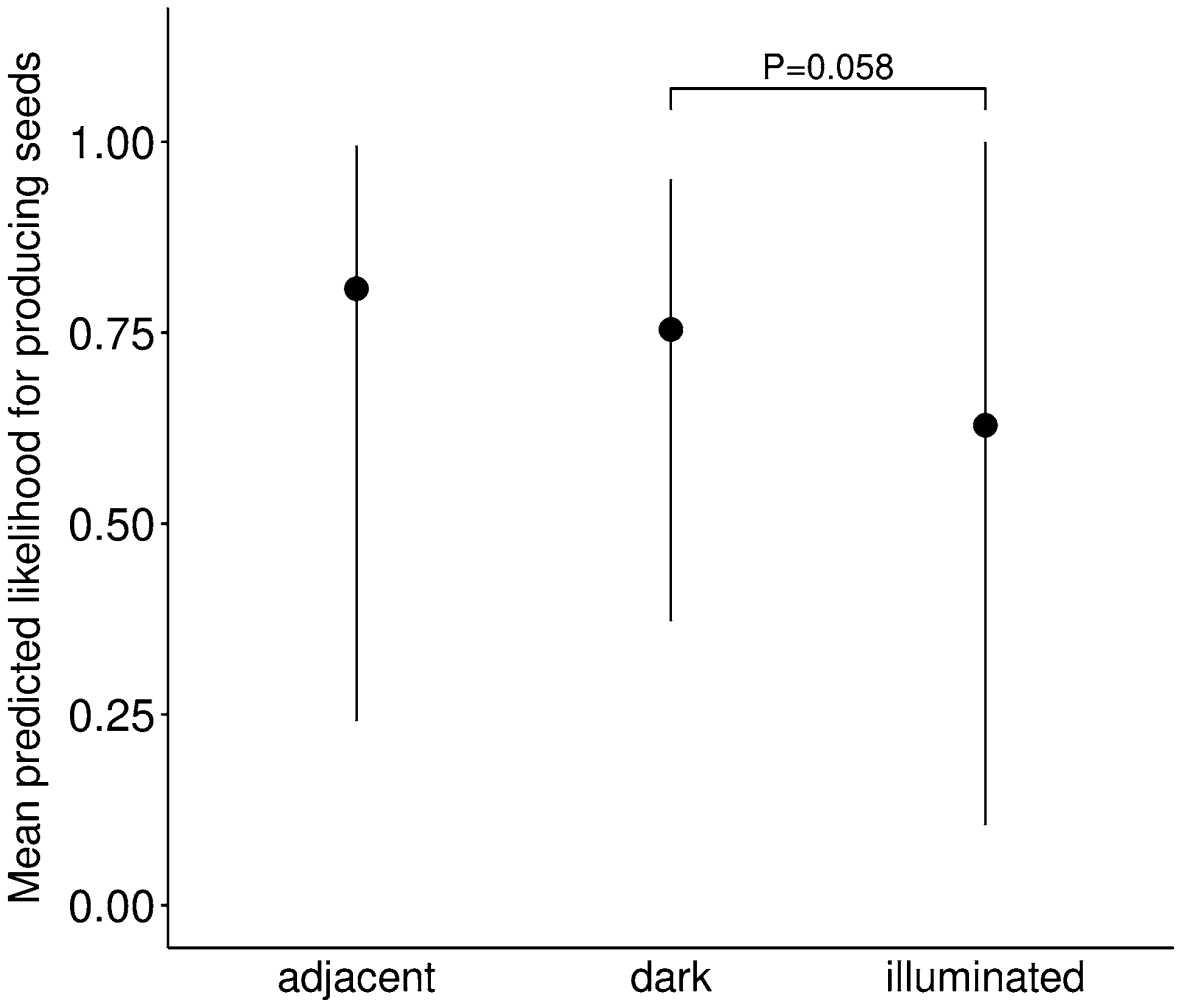
Effects of the light treatment on the likelihood of a subset of capsules to produce seeds in *Epilobium angustifolium*. Dark: plants exposed on dark control sites; illuminated: plants exposed on illuminated sites; adjacent: plants exposed on a dark site but adjacent to the illuminated site (N = 240). Mean of model predicted values ± 95% confidence interval, as well as significance levels P ≤ 0.1 are shown.

Two proxies of reproductive output were measured for both plant species, namely the likelihood of a subset of capsules to produce seeds and the number of seeds per capsule (see methods). For *S. latifolia*, there was no effect of the light treatment (three levels: illuminated, dark, adjacent) on the likelihood to produce seeds. For *E. angustifolium* there was a trend for a reduced likelihood to produce seeds in plants exposed on the illuminated sites compared to the dark sites (estimate (estim.) = − 1.37, standard error (SE) = 0.726, z = − 1.9, degrees of freedom (d.f.) = 234, P = 0.058, Table 1, Fig. 2), while no effect was found in plants exposed on the adjacent sites compared to the dark sites (Table 1, Fig. 2). For both plant species there was no effect of the treatment on the number of produced seeds.

**Table 1:** Effects of the light treatment in field experiment (dark: plants exposed on dark control sites; illuminated: plants exposed on the bright side of illuminated sites; adjacent: plants exposed on the dark side of illuminated sites) on (a) the likelihood of a subset of capsules to produce seeds in *Epilobium angustifolium*, (b) the plant infection rate of *Silene latifolia* and (c) plant relative reproductive rate.

	Estimate	SE	z value	P value
**(a)**				
Dark (intercept)	0.43	0.656	0.7	0.512
Adjacent	− 0.03	0.922	0.0	0.975
Illuminated	− 1.37	0.726	− 1.9	**0.058**
Total flowers	0.03	0.009	3.0	**0.003**
**(b)**				
Adjacent (intercept)	0.24	0.536	0.4	0.659
Illuminated	− 1.14	0.363	− 3.1	**0.002**
Dark	− 1.90	0.576	− 3.3	** < 0.001**
Total flowers	− 0.01	0.007	− 1.8	**0.077**
**(c)**				
Adjacent (intercept)	− 0.74	0.464	− 1.6	0.109
Illuminated	0.69	0.371	1.9	**0.063**
Dark	1.04	0.499	2.1	**0.037**
Total flowers	− 0.00	0.006	0.0	0.977

*P* ≤ 0.1 are presented in bold.

**Figure 3:**
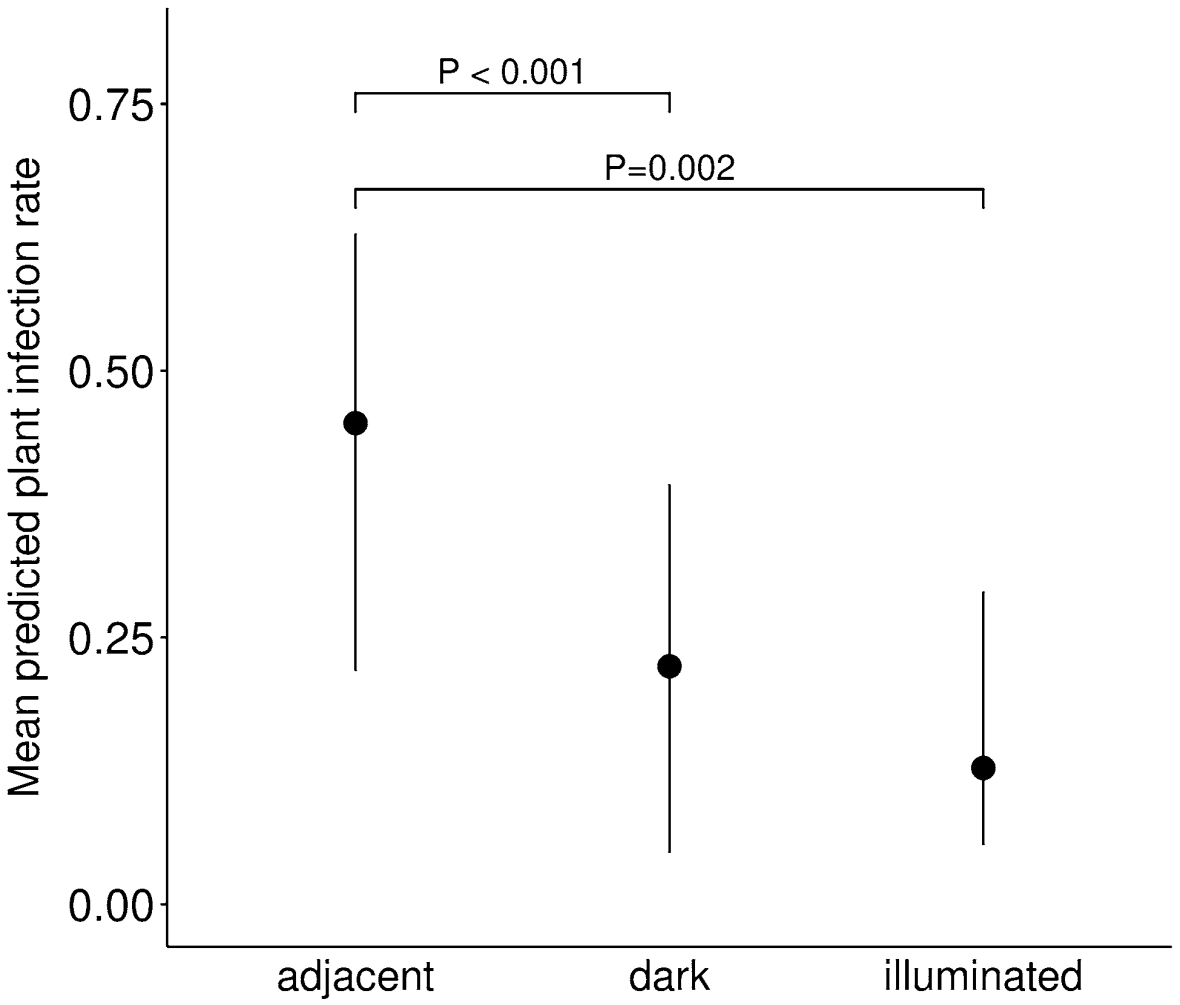
Effects of the light treatment on *Silene latifolia* plant infection rate. Dark: plants exposed on dark control sites; illuminated: plants exposed on illuminated sites; adjacent: plants exposed to a dark site but adjacent to the illuminated site (N = 47). Mean of model predicted values ± 95% confidence interval, as well as significance levels P ≤ 0.1 are shown.

**Figure 4:**
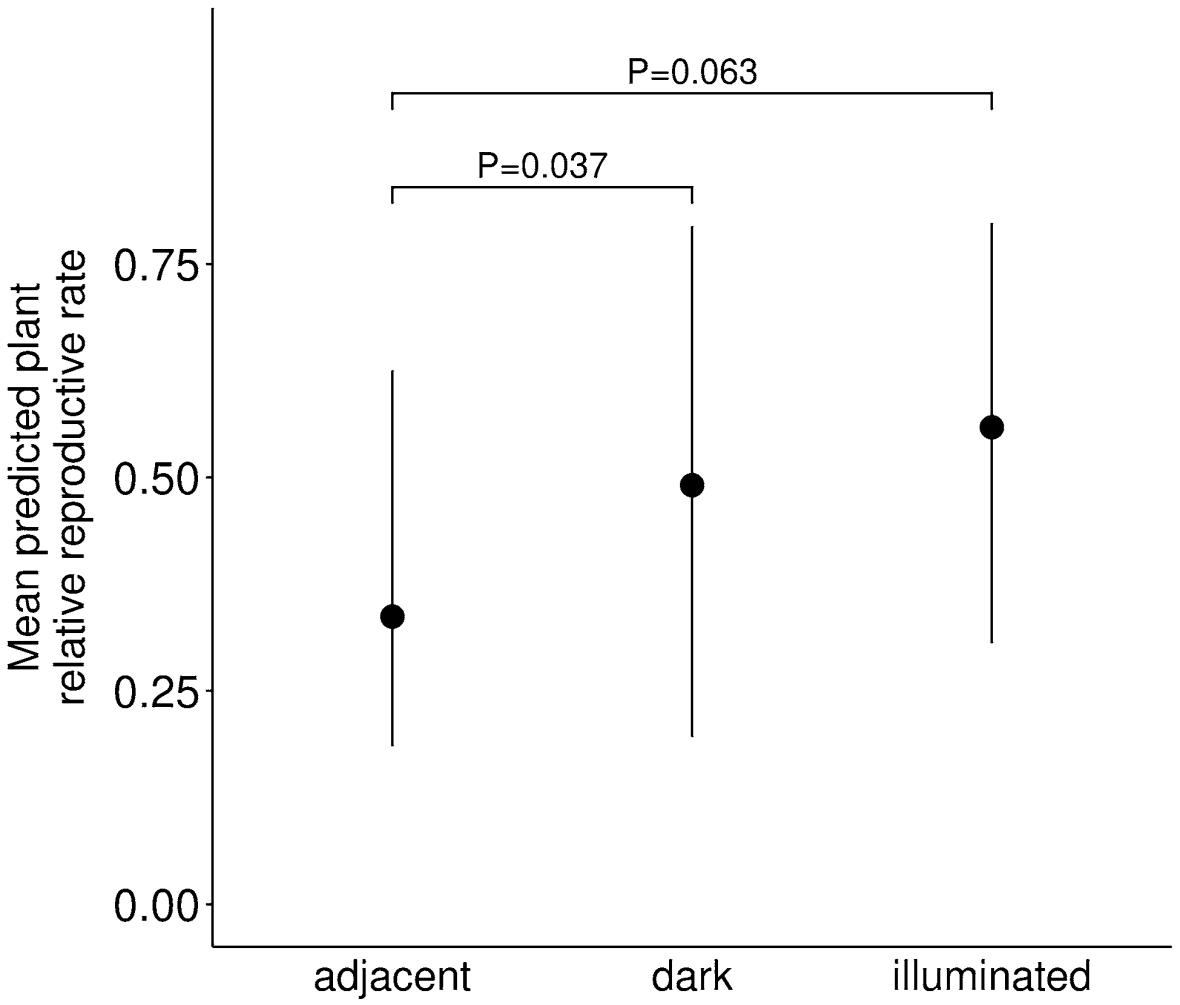
Effects of light treatment on *Silene latifolia* plant relative reproductive rate. Dark: plants exposed on dark control sites; illuminated: plants exposed on illuminated sites; adjacent: plants exposed on dark sites but adjacent to the illuminated sites (N = 47). Mean of model predicted values ± 95% confidence interval, as well as significance levels P ≤ 0.1 are shown.

The effect of ALAN on pre-dispersal seed predation of *S. latifolia* was analysed by estimating the rate of fruit infections by *H. bicruris* (see methods). There was a decreased infection rate in plants that were exposed on dark sites compared to the sites adjacent to the directly illuminated sites (estim. = − 1.90, SE = 0.576, z = − 3.3, d.f. = 42, P < 0.001) and illuminated sites, respectively (estim. = − 1.14, SE = 0.363, z = − 3.1, d.f. = 42, P = 0.002, Table 1, Fig. 3). Infection rate was similar of plants exposed on illuminated sites compared to plants exposed on dark sites (estim. = 0.76, SE = 0.521, z = 1.46, d.f. = 42, P = 0.145). Finally, we also quantified the combined effect of mutualistic interactions of *S. latifolia* with its pollinators and of its antagonistic interaction with its main seed predator *H. bicruris*. We thus calculated the relative reproductive rate, defined as the amount of fruit capsules not attacked by *H. bicruris* relative to (divided by) the total amount of exposed flowers. Plant relative reproductive rate was significantly increased in plants exposed on dark sites compared to plants exposed on sites adjacent to illuminated sites (estim. = 1.04, SE = 0.499, z = 2.1, d.f. = 42, P = 0.037, Table 1, Fig. 4). There was a trend for an increased plant relative reproductive rate of plants exposed on illuminated sites compared to plants exposed on sites adjacent to illuminated sites (estim. = 0.69, SE = 0.371, z = 1.9, d.f. = 42, P = 0.063, Table 1, Fig. 4).

### Effect on plant-flower visitor interactions and egg laying behaviour

**Figure 5:**
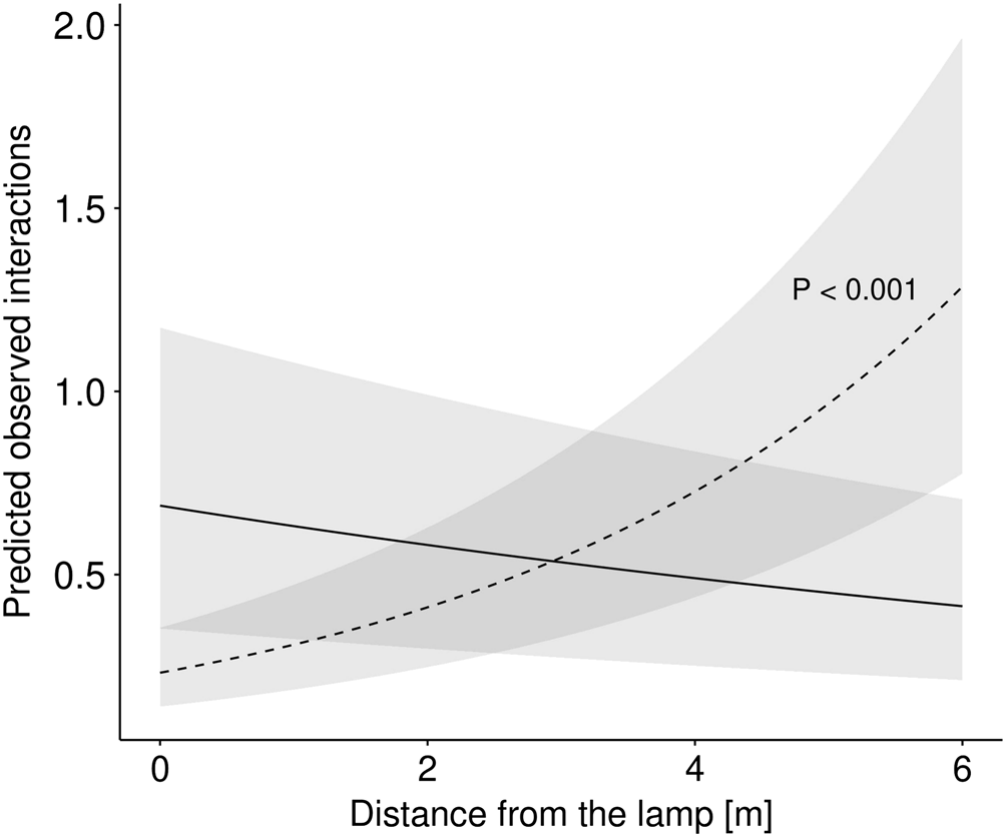
Effects of the light treatment on the number of interactions between *Silene latifolia* and *Hadena bicruris*. Solid line (treatment dark) and dashed line (treatment illuminated) show the predicted observed interactions as a function of distance from the lamp, while shaded areas show 95% confidence interval (N = 108). Significance levels P ≤ 0.1 are shown.

**Figure 6:**
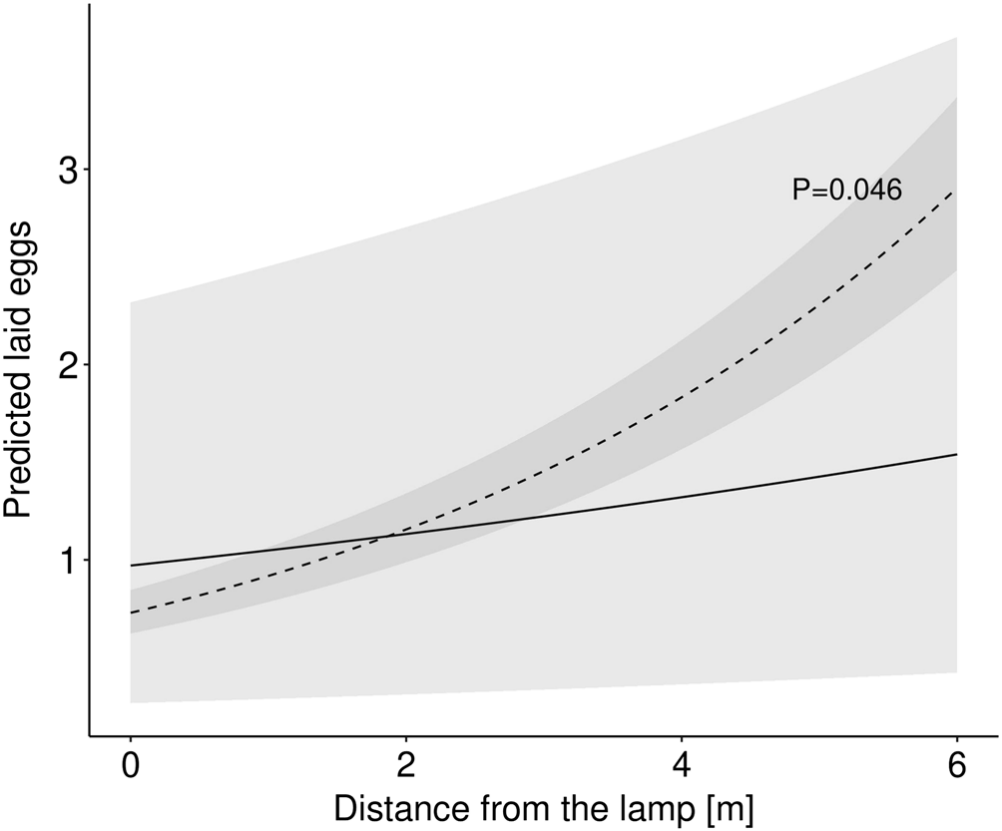
Effects of the light treatment on the number of eggs laid by *Hadena bicruris* on and into the flowers of *Silene latifolia*. Solid line (treatment dark) and dashed line (treatment illuminated) show the predicted observed interactions as a function of distance from the lamp, while shaded areas show 95% confidence interval (N = 108). Significance levels P ≤ 0.1 are shown.

To test for the impact of ALAN on the number of interactions of *H. bicruris* with the flower of *S. latifolia* and the egg laying behaviour of the moth under controlled conditions, we used three independent corridors (8 m long, 1 m wide, 2.8 m high) along which we either established a light-intensity gradient starting from a focal lamp and corresponding to the light conditions of the field experiment (further referred to lit corridor) or which we kept as a dark control by installing a lamp that was switched off (further referred to as dark corridor). In the corridors we then repeatedly offered flowers of *S. latifolia* to egg-laying females of *H. bicruris*, recorded the number of interactions, and the number of eggs laid (see methods). The number of visits to flowers of *S. latifolia* was significantly reduced in lit corridors compared to dark corridors (estim. = − 1.22, SE = 0.522, z = − 2.3, d.f. = 101, P = 0.020, Table 2). Furthermore, the number of flower visits increased significantly with distance from the lamp in the lit corridors (estim. = 0.37, SE = 0.104, z = 3.6, d.f. = 101, P < 0.001, Table 2, Fig. 5), but not in the dark corridors (estim. = − 0.08, SE = 0.075, z = − 1.1, d.f. = 101, P = 0.263, Table 2). Distance from the releasing point to the flowers had a significant, negative effect on the number of activity events in the dark corridors (estim. = − 0.20, SE = 0.096, z = − 2.1, d.f. = 101, P = 0.034), but had no effect in the lit corridors (estim. = 0.05, SE = 0.127, z = 0.4, d.f. = 101, P = 0.716). Furthermore, no fatal flight to light behaviour was observed in the lit corridors (i.e., no moths circling around the lit lamp until exhaustion). Consistent with the amount of flower visits of the moth to *S. latifolia*, we found a negative trend for the number of eggs laid on and into *S. latifolia* flowers in lit corridors compared to dark corridors (estim. = − 1.17, SE = 0.622, z = − 1.9, d.f. = 100, P = 0.060, Table 2). Also, there was a positive effect of the distance from flowers of *S. latifolia* to the light source on the number of eggs laid in lit corridors (estim. = − 0.24, SE = 0.123, z = 1.9, d.f. = 100, P = 0.046, Table 2, Fig. 6). On the other hand, there was not such effect in dark corridors (estim. = 0.06, SE = 0.094, z = 0.6, d.f. = 100, P = 0.557, Table 2). There was a significant, negative effect of the distance from the releasing point to the flowers on the number of eggs laid in dark corridors (estim. = − 0.32, SE = 0.115, z = − 2.7, d.f. = 100, P = 0.006, Table 2), but not in lit corridors (estim. = 0.24, SE = 0.146, z = 1.7, d.f. = 100, P = 0.097, Table 2). Finally, there was no effect of ALAN on the total number of eggs laid in dark corridors compared to lit corridors (estim. = 0.15, SE = 0.239, z = 0.6, d.f. = 24, P = 0.520), excluding a paralyzing effect of ALAN as possible explanation for the low number of eggs laid close the burning lamp.

**Table 2:** Effects of the light treatment in laboratory experiment (dark: LED lamp off; illuminated: LED lamp on inducing a light gradient) on (a) the number of interactions between *Silene latifolia* and *Hadena bicruris* and (b) the number of eggs laid by *H. bicruris* on and into the flowers of *S. latifolia* and moth paralysis when released close to the lamp was also tested (c).

	Estimate	SE	z value	P value
**(a)**				
Dark (intercept)	0.25	0.372	0.7	0.497
Illuminated	− 1.22	0.522	− 2.3	**0.020**
Dark: distance from lamp	− 0.08	0.075	− 1.1	0.263
Dark: distance from releasing point	− 0.20	0.096	− 2.1	**0.034**
Illuminated: distance from lamp	0.37	0.104	3.6	**< 0.001**
Illuminated: distance from releasing point	0.05	0.127	0.4	0.716
**(b)**				
Dark (intercept)	0.57	0.471	1.2	0.227
Illuminated	− 1.17	0.622	− 1.9	**0.060**
Dark: distance from lamp	0.06	0.094	0.6	0.557
Dark: distance from releasing point	− 0.32	0.115	− 2.7	**0.006**
Illuminated: Distance from lamp	0.24	0.123	1.9	**0.046**
Illuminated: distance from releasing point	0.24	0.146	1.7	0.097
**(c)**				
Dark (intercept)	1.78	0.198	9.0	**< 0.001**
Illuminated	0.15	0.239	0.6	0.520

*P* ≤ 0.1 are presented in bold.

## Discussion

There was a trend for a negative effect of direct illumination at night on the reproductive output (likelihood of selected fruit capsules to produce seeds) of one of the two plant species (*E. angustifolium*). This supports findings of a previous study that found a negative effect of direct illumination on the fruit set of *Cirsium oleraceum* (Asteraceae)^12^. Also, it suggests that direct illumination of plants can disrupt the pollination service at night, which cannot be compensated by pollinators active during daytime. On the other hand, the absence of a negative effect of direct illumination on the reproductive output of *S. latifolia* is consistent with findings of another recent study^23^ and may be explained by a lack of complementarity of night-active and day-active pollinators for this plant species, i.e. the diurnal pollinators seem to be able to compensate the reduced pollination at night^12,24^. Alternatively, the latter might be explained by the fact, that the main pollinator *H. bicruris* seems to be able to cope with artificial light at night, as the results from our experiment under controlled conditions showed. Thus, even though *H. bicruris* preferred to visit the flowers which experienced as low light intensity as possible, it could be that the moth might have pollinated them equally well as the plants were exposed in the field for almost a month and the *S. latifolia* was not very abundant in the area.

Interestingly, the effect of ALAN on fruit infection rate of *S. latifolia* caused by the moth seed predator, *H. bicruris*, was highest on sites adjacent to directly illuminated sites. This also led to the lowest relative reproductive rate of plants on those sites. The underlying mechanism for these results was most likely a change of the oviposition behaviour of the seed predator in the presence of ALAN: the controlled experiment in the laboratory showed that females of the seed predator flew away from the light source to lay eggs on plants exposed to as low light intensity levels as possible. Thus, it could be that in the field, the females also flew away from the illuminated site and preferred to lay eggs on plants that were not exposed to direct illumination. This suggests that ALAN can alter interactions and ecosystem functioning even beyond the edge of the directly illuminated area.

One reason, why *H. bicruris* searched dark oviposition sites could be that they might provide minimal risk of offspring parasitoidism, for example by *Microplitis tristis* (Hymenoptera: Braconidae)^25,26^. Even though *M. tristis* is considered to be diurnal, a dark location might be advantageous as a recent study found an extension of a day-active parasitoid of aphids due to ALAN^27^. Alternatively, dark locations might be advantageous for oviposition as host plant quality might be changed by direct illumination, thereby lowering the fitness of the larvae^28^.

In contrast to what we found in the laboratory experiment, *S. latifolia* plant infection rate was similar in plants exposed on the illuminated sites compared to those exposed on the dark sites. One reason for these findings could be that street lamps installed in otherwise dark study regions are likely to be visible from a long distance by flying insects^1^ such as *H. bicruris*. It is therefore possible that *H. bicruris* were attracted to the illuminated sites from long distances, thereby leading to a locally increased density. At the same time our laboratory experiment showed that *H. bicruris* was able to interact with *S. latifolia* despite ALAN, but that it preferred dark locations. Together this might have led to significantly higher infection rate in adjacent sites. We suggest that, as a consequence of local high density of *H. bicruris*, once locations suitable for oviposition on sites adjacent to the illuminated sites were all exploited, the moths might have started visiting plants on the illuminated sites leading to infection rates similar to those recorded on dark control sites. It is important to note that the plants were exposed for almost four weeks in the field and *S. latifolia* was not very abundant in the study area, potentially leading to intraspecific competition for oviposition sites.

Our results furthermore show that ALAN can condition the outcome of a nursery pollination system, where the adult pollinator oviposits in the flower and the hatching larvae feeds on the developing seeds. Pollination nursery systems are located on a *continuum* with antagonism (seed predation) on the one end and mutualism (pollination) on the other: in the considered system ALAN shifted the position of the relationship toward antagonism in the area adjacent to an illuminated location. It is known that the outcome of mutualisms (and of other species interaction types) is conditional to environmental gradients and other contingencies^29–32^. However, it is to the best of our knowledge, the first evidence that ALAN can shift this *continuum*. Given the large extension of ALAN, it remains an open question what the implications of our findings for the stability of the *Silene*-*Hadena* nursery pollination system are.

In sum, we showed that ALAN can affect ecosystem functioning in dark areas surrounding an area which is directly illuminated by artificial light. Thus, ALAN might affect biodiversity, species interactions and ecosystem functioning at a much larger spatial scale than previously thought.

## Methods

### Field experiment

#### Study design

In 2017, eight unmanaged meadows were selected in the Prealps of Switzerland. This region has low levels of light emission with a radiance lower than 0.25 × 10^-9^ W sr^-1^ cm^-2^ (data from https://www.lightpollutionmap.info). Meadows had an average linear distance to the nearest site of 1.45 ± 0.34 km. The sites were located in the middle of the meadows on as homogenous vegetation as possible, so that there was no influence by elements like bushes or forest edges. The most abundant and widespread plant species on the meadows was *Cirsium oleraceum* (Asteraceae), followed by other plant species being abundant but not present on all sampling sites: *Angelica sylvestris* (Apiaceae), *Eupatorium cannabinum* (Asteraceae), *Erigeron annuus s.l.* (Asteraceae) and *Filipendula ulmaria* (Rosaceae). On four out of the eight meadows we experimentally installed a LED street lamp (Schréder GmbH, type: AMPERA MIDI 48 LED, colour temperature: neutral white (4,000 K), nominal LED flux: 6,800 lm) on 6 m high poles. Street lamps were installed on one side of the meadows, which resulted in an experimental set-up, where during nighttime a part of the meadow was illuminated by a cone of light. The part of the meadow further from the experimentally set-up street lamp was not illuminated and its darkness corresponded to the darkness measured on the control meadows that had no artificial light source in the vicinity. In other words, the four meadows were divided by artificial light into two parts, one directly illuminated by the lamp and the other being dark but adjacent to the illuminated part. Subsequently, we refer to the two parts as two sites, even though they were part of the same meadow, i.e., the illuminated part is further referred to as illuminated site, the dark part adjacent to the illuminated part as adjacent site (see Fig. 1). It is important to notice, that the street lamp was experimentally established, i.e., there was no systematic bias in terms of other landscape structures (such as roads, forest edges or hedges) where the illuminated part of the meadow was, adjacent, respectively. Thus, landscape structures that were different between the illuminated and dark part of a meadow potentially influenced the results in a non-systematic way and increased variance, but did not create a systematic bias. The remaining four meadows were left completely dark (further referred to as dark control sites), but they were equipped with a fake street lamp to provide comparable conditions. Light intensity on illuminated sites followed a negative exponential curve as function of the distance from the lamp dropping from 75.73 ± 1.54 lx just under the lamp (< 2 m) to 2.67 ± 0.19 lx 10 ± 1.0 m away. We considered as the adjacent site the locations where the measured light intensity was equal or lower to 2.82 × 10^−3^ lx, corresponding to the third quartile of the light intensities on dark sites. Light measurements were performed at least two hours after sunset and in the absence of moon, using an universal photocurrent amplifier with computer interface (by Czibula and Grundmann GmbH, https://www.photo-meter.com), always keeping the sensor at 70 cm of height (the average flowers height) and pointing upward. All sites were located at least 100 m away from permanent light sources and 500 m away from major light sources, such as illuminated sport grounds.

#### Phytometers

The two plant species, *Silene latifolia* (Caryophyllaceae) and *Epilobium angustifolium* (Onagraceae), were selected as phytometers because they were present in the study area and need animal pollination for maximal reproductive output (*E. angustifolium*: https://doi.org/10.1111/j.1366-9516.2004.00106; *S. latifolia*: dioecious). Also, both species are known to be visited by nocturnal and diurnal pollinators^33–35^. Furthermore, *S. latifolia* is involved in nursery pollination with the nocturnal moth *Hadena bicruris* (Noctuidae): while pollinating female flowers of *S. latifolia*, females of *H. bicruris* often lay one or more eggs into the flowers. After hatching their larvae feed on the developing seeds of *S. latifolia*^36^. Contrarily to other nursery pollination systems^37,38^, the considered one is not mandatory and *S. latifolia* can also be pollinated by other insects^33^. However, in the European native range species of the genus *Hadena* are considered to be the main pollinators^22^. Between March and April 2017 we sowed plants and grew them in the greenhouse. We applied once a standardized amount of fertilizer (Wuxal universal fertilizer by Maag, https://www.maag-garden.ch) about six weeks after sowing. When buds where about to bloom we brought the plants to the field and randomly assigned them to our test sites. Doing so, special attention was devoted to small scale topographic elements such as bushes, fences and distances from forest edge (when present), with the goal to provide comparable conditions to the phytometer locations on our sites.

#### Experimental set-up of phytometers

We exposed a total of 97 potted plants (47 *S. latifolia* and 50 *E. angustifolium*) on the sites: 26 plants (13 *S. latifolia* and 13 *E. angustifolium*) were equally distributed on the four dark control sites (mean light intensity measured on the phytometers: 4.0 × 10^−3^ ± 4.4 × 10^−4^ lx, min. 0.7 × 10^−4^ lx, max. 6.4 × 10^−3^ lx), 51 plants (25 *S. latifolia*, 26 *E. angustifolium*) on illuminated sites (mean light intensity measured on the phytometers: 16.1 ± 2.8 lx; min. 4.1 × 10^−3^ lx, max. 58.3 lx), and 20 plants (9 *S. latifolia* and 11 *E. angustifolium*) on sites adjacent to illuminated sites (mean light intensity measured on the phytometers: 2.1 × 10^−3^ ± 6.7 × 10^−5^ lx; min. 1.8 × 10^−3^ lx, max. 2.7 × 10^3^ lx). Figure 6 shows schematically the experimental set-up of phytometers.

As *S. latifolia* is dioecious, we provided male plants as pollen donors (one per control site, three per illuminated and adjacent site) at least 20 m apart from each other to avoid dependencies. For the same reason, females *S. latifolia* and hermaphrodite *E. angustifolium* were kept at least 10 m apart from conspecifics. On illuminated sites pollen donors were evenly distributed while accounting for the topography of the terrain and the proximity with females of *S. latifolia*.

Depending on the weather, plants were watered every three days, and we monitored them until most of their flowers were withering or fructifying. The average exposition time was, in days, 42.1 ± 1.1 for *E. angustifolium* and 24.1 ± 0.3 for *S. latifolia*. The latter phytometer could have been exposed to experimental conditions longer since it kept producing flowers. Nevertheless we removed them from the field before *H. bicruris* larvae started to emerge from the infected fruit capsules to attack other healthy capsules (secondary infections^39,40^).

### Estimation of reproductive output and pre-dispersal seed predation

After bringing back the plants from the field, we let them fully develop the fruit in climate chambers. To prevent secondary infections of *S. latifolia*, all capsules were daily checked for the presence of *H. bicruris* (hole(s) on the capsule surface and presence of frass), and infected capsule were immediately bagged. Only flowers that opened and withered during exposure to experimental conditions in the field were included in the analysis, hereafter referred to “exposed flowers”.

Two measures of reproductive output were quantified. First, of each plant individual the number of fully developed seeds of a maximum of five randomly selected exposed flowers that produced a capsule (and were therefore susceptible to being successfully pollinated; hereafter called “selected capsules”) was counted. In *S. latifolia* only uninfected capsules were taken as selected capsules. This resulted in the analysis of the seed set of 240 and 199 capsules from *E. angustifolium* (Onagraceae) and *S. latifolia* (Caryophyllaceae), respectively. Based on these seed counts we estimated the likelihood of selected capsules to produce seeds, and we used it as a proxy for pollination success (yes/no). Because *S. latifolia* is dioecious we considered a flower as successfully pollinated if at least one fully developed seed was found in the capsule. In *E. angustifolium* self-fertilization is efficiently reduced by protandry^41^ but not completely avoided. Based on seed counts from 16 potted *E. angustifolium* that were placed on control sites and caged to prevent flower visitations, we estimated self-pollination rate (mean 3.37 ± 1.27 developed seed counted) and we considered a flower as successfully pollinated if four or more fully developed seeds were found in the capsule.

*Epilobium angustifolium* produces racemes where flowers toward the base of the raceme bloom and wither before those toward the top. As a consequence, flowers and fruit capsules on different states of development are available at the same time. To avoid bias related to that, we randomly chose the five selected fruit capsules within the raceme. If more than one raceme was available on a single plant we chose the biggest one, i.e. bearing bigger and more numerous flowers.

To assess the output of an antagonistic interaction, we estimated pre-dispersal seed predation by *H. bicruris*. Plant infection rate was defined for each *S. latifolia* plant individual as the number of capsules primarily attacked by *H. bicruris* larvae on the total amount of flowers that developed a capsule. Finally, we also quantified the combined effect of mutualistic interactions of *S. latifolia* with its pollinators and of its antagonistic interaction with its main seed predator *H. bicruris*. We thus calculated the relative reproductive output, which was the amount of fruit capsules not attacked by *H. bicruris* over the total amount of exposed flowers. We took a relative measure of reproductive rate to account for the variation of number of flowers produced among *S. latifolia* plant individuals.

### Response of flower visitation and egg laying behaviour to ALAN

To test for the impact of ALAN on the number of interactions of the moth with the flower and its egg laying behavior under controlled conditions, we set up an experiment with flowers of *S. latifolia* and females of *H. bicruris* under controlled conditions. We split lengthwise a room to establish three independent corridors (8 m long, 1 m wide, 2.8 m high) using black plastic sheets and we induced a light-intensity gradient by placing a lamp (Osram E27, 4,000 K, 4 W, 470 lm) at 1.3 m height at one end of the corridors. In each corridor flowers of *S. latifolia* were offered to a female of *H. bicruris* (respectively grown and bred under standardized conditions, in greenhouse and climate chamber; light day/night: 14/10 h, temperature day/night: 22/18 °C) at four positions (position 1: 0 m distance from the lamp; position 2: 2 m; position 3: 4 m; position 4: 6 m). This corresponded to 90.3 ± 0.3 lx, 10.7 ± 0.7 lx, 2.0 ± 0.0 lx and 1.0 ± 0.0 lx, respectively. Interactions between the flowers of *S. latifolia* with females of *H. bicruris* were filmed (Sony Handycam DCR-SR200, 4.0 mega pixels). Egg laying behaviour in response to light was recorded by counting the number of eggs laid over the course of one night under different light intensities. For each experimental replicate we released always one single moth. We used moths from wild larvae collected in the area of the field sites and bred according to an established methodology^25^. When adults emerged we paired couples of one male and one female and we used females for the experiment as soon as they started to lay fertilized eggs. During all development stages *H. bicruris* were kept in climate chambers at 25/15 °C 14/10 h L/D^25^. For each experimental replicate we placed at each position a new pair of female flowers that started to bloom 2.9 ± 0.1 days earlier. We chose this flower age as the scent emission of the flowers should then be maximal^42^. Flowers were clipped just below the receptacle and the pedicel stump fixed between the border of a tube filled with water and its foam plug. Experiment repeats started at 19:00 in synchrony with the simulated sunset in the climate chamber, when a moth was released at a random location in one of the corridors and randomly assigned to a lit corridor (lamp switched on) or a dark corridor (lamp switched off) treatment. Repeats ended the following morning when we collected moths, flowers and video cameras. Footages from cameras were imported to a computer and analyzed by a Matlab^43^ program (see Supplementary material 1) comparing each frame of a footage with the preceding one and providing a quantitative measure of difference between frames. We thereby were able to automatically detect movements of moths on the flowers and around the lamp. Also, flowers were carefully dissected to check for eggs.

We ran 27 replicates, 11 in the dark corridor and 16 lit corridor. In total 216 *S. latifolia* flowers were checked for oviposition (88 of the dark corridor and 128 of the lit corridor). Twenty-two female moths were used, each 9 were used once for a dark corridor, for a lit corridor, respectively. Two female moths were used twice for a lit corridor, and one was used first for a dark corridor and subsequently for a lit corridor. Finally, one moth was used once for a dark corridor and subsequently twice for a lit corridor. Each replicate was filmed with the above mentioned video cameras and set up and a total of 1,400 h of footages was analyzed.

In order to avoid a bias of the moth flying to the nearest flower after release, we defined three releasing points: 1 m behind position 4 (opposite direction from the lamp), between position 2 and 3 and beside the lamp, about 0.3 m from position 1.

### Statistical analysis

To test for the effect of ALAN on the considered dependent variables we ran generalized linear mixed-effects models (glmmTMB function from the R^44^ package glmmTMB^45^). We checked all models for overdispersion by including an observation-level random factor (as many levels as observations) into the model and comparing it to a version of the same model without this additional random factor. When the observation-level random factor significantly improved the model, it was retained in the model.

#### Number of seeds per capsule

The effect of ALAN on the amount of developed seeds per selected capsule was analyzed separately for *S. latifolia* and *E. angustifolium* assuming a Poisson distribution of the data. Light treatment (three levels: dark, adjacent and illuminated) was included as a fixed factor and the total number of flowers per plant as a co-variable. Plant individual was nested within site and included as random factor.

#### Likelihood of selected capsules to produce seeds

The effect of ALAN on the likelihood of selected capsules to produce seeds was analyzed separately for *S. latifolia* and *E. angustifolium* using a binary variable (pollinated versus non-pollinated) and assuming a Binomial distribution of the data. Light treatment (three levels: dark, adjacent and illuminated) was included as a fixed factor and the total number of flowers per plant as a co-variable. Plant individual was nested within site and included as random factor.

#### Plant infection rate in *S. latifolia*

The effect of ALAN on plant infection rate was analyzed using a two columns matrix with the number of infected fruit capsules in the first column and the number of not infected fruit capsules as dependent variable and assuming a Binomial distribution of the data. Light treatment (three levels: dark, adjacent and illuminated) was included as a fixed factor and the total number of flowers per plant as a co-variable. Site was included as random factor.

#### Plant relative reproductive rate in *S. latifolia*

The effect of ALAN on plant relative reproductive rate was analyzed using proportion data and the same model structure as the previous analysis. The dependent variable was a two columns matrix with the number of fruit capsules not attacked by *H. bicruris* in the first column and the total number of flowers exposed to the experimental conditions in the second.

#### Flower visits

The effect of ALAN on the number of flower visits of *H. bicruris* to flowers of *S. latifolia* was analyzed assuming a Poisson distribution of the data. The model included treatment (two levels: dark vs. illuminated) as fixed factor, distance between lamp and flowers and distance between releasing point and flowers as continuous variables, and the interactions between the treatment and the continuous variables. The random factor was moth individual (22 levels).

#### Proportion of eggs laid

The effect of ALAN on the amount of eggs laid by females *H. bicruris* on flowers of *S. latifolia* was analyzed using the same model structure as described for the number of visits.

#### Test for paralyzing effect of light

We analyzed whether uneven amounts of eggs laid on flowers located in different positions was due to the fact that females of *H. bicruris* released close to the lamp were paralyzed by the high light intensity. To do so we pooled the data of each replicate to get the total of laid eggs and we analyzed it. We assumed a Poisson distribution of the data and included treatment (two levels: dark and light) as fixed factors. The random factor was moth individual (22 levels).

All analysis were performed using R^44^.

## Supplementary information

Supplementary file1

## Data Availability

The datasets generated and analysed during the current study are available from the corresponding author on reasonable request.
