# Supplementary material for: Artificial light at night can modify ecosystem functioning beyond the lit area
Source: Sci Rep. 2020 Jul 17;10:11870. doi: 10.1038/s41598-020-68667-y (PMC7368033; doi:10.1038/s41598-020-68667-y)
Supplement: Supplementary file 1 — Supplementary file1 [file 41598_2020_68667_MOESM1_ESM.pdf]

Supplementary material: Matlab code

Title: Artificial light at night can modify ecosystem functioning beyond the lit area

Authors: Simone Giavi[1,4], Sina Blösch[1,2], Guido Schuster[3], Eva Knop*[4,5]

Affiliations:

[1] University of Bern, Institute of Ecology and Evolution, Baltzerstr. 6, 3012 Bern, Switzerland

[2] Bernese School of Agricultural, Forest and Food Sciences HAFL, Länggasse 85, 3052 Zollikofen, Switzerland

[3] University of Applied Sciences of Eastern Switzerland, Department of Electrical Engineering, Oberseestrasse 10, 8640 Rapperswil, Switzerland

[4] Agroscope, Agroecology and Environment, Reckenholzstrasse 191, Zürich 8046, Switzerland

[5] University of Zürich, Department of Evolutionary Biology and Environmental Studies, Winterthurerstr. 190, 8057 Zürich, Switzerland

Corresponding author contact: eva.knop@ieu.uzh.ch

Scritp 1: calculateandstore.m

```
function calculateandstore2(name)

tic

m=mmread(strcat(name,'.mp4'),[],[0 1],false,true);

maxseconds=m.totalDuration;OverallFrame=0;


for second = 0:50:maxseconds-60 %make sure it works

   toc

   second

   tic

    m=mmread(strcat(name,'.mp4'),[],round([second second+50]),false,true);

    if second==0

        lastFrame=m.frames(1).cdata;

    end


    for FrameNumber=1:size(m.frames,2) %careful there are compression artefacts

        OverallFrame=OverallFrame+1;


        if FrameNumber==size(m.frames,2)

            lastFrame=m.frames(FrameNumber).cdata;

        end


        if FrameNumber==1

            delta=double(rgb2gray(m.frames(FrameNumber).cdata-lastFrame));

        else

            delta=double(rgb2gray(m.frames(FrameNumber).cdata-m.frames(FrameNumber-
1).cdata));

        end
```

```
        STDImage(OverallFrame)=std(delta(:));

    end

end
clear m
save(name)
```

Scritp 2: displayResults2.m

```
%clear all;

%close all;


%name='HB740_2018-04-14';

%cd(name) %change into that directory


%read the lamp file if it exists - it get the index 1 but is at

%position 0


temp=strcat('Lamp_',name1);

if exist(strcat(temp,'.mat'),'file')

    load(temp) %load the lamp file info

    fps(1)=OverallFrame/maxseconds; %frame rate

    STDk=medfilt1(STDImage,25)'; %supress compression artefacts spikes that come
every 10 seconds

    STDk=STDk(25:end); %supress a second at the start, often incorrect

    %STD{1}=STDk-median(STDk);

    STD{1}=STDk;

else

    fps(1)=NaN;

    STD{1}=NaN;

end
```

```
%read the position files, if they exist
prename='PosX_';
for k=1:4
    prename(4)=num2str(k);
    temp=strcat(prename,name1);
    if exist(strcat(temp,'.mat'),'file')
        load(temp)
        fps(k+1)=OverallFrame/maxseconds;
        STDk=medfilt1(STDImage,25)';
        STDk=STDk(25:end); %supress a second at the start, often incorrect
        %STD{k+1}=STDk-median(STDk);
        STD{k+1}=STDk;
    else
        fps(k+1)=NaN;
        STD{k+1}=NaN;
    end
end

%create the 3D plot with the lamp at +2m, postion 1 at 0 etc.
fig1=figure(1);
for k=1:5
```

```
    plot3( -2*(k-2)*ones(size(STD{k})),(1:length(STD{k}))/(fps(k)*60),STD{k}); hold
on

end

hold off

xlabel('Position [m]'); ylabel('Time [min]'); zlabel('Activity');title(name1,
'interpreter', 'none');

grid on; axis tight; a=axis;

axis([-7 3 -5 a(4) -1 7]); yticks(0:15:1000);

%write it as a pdf file

orient(fig1,'landscape')

print(strcat(name1,'_3D'),'-fillpage','-dpdf');


%create the 2D plot with pos 4 on top (where the insect is

%released) and the lamp at the bottom

fig2=figure(2);

for k=1:5

    ax(k)=subplot(5,1,6-k); plot((1:length(STD{k}))/(fps(k)*60),STD{k});

    ylabel('Activity');

    if k==1 %only write the time label once

        xlabel('Time [min]');

        temp=strcat('Lamp_',name1);

    else

        prename(4)=num2str(k-1);
```

```
        temp=strcat(prename,name1);
    end
    title(temp,'interpreter', 'none');
    xticks(0:15:1000);  axis([-5 a(4) -1 7]);
end
linkaxes(ax);

%print the 2D graph as a pdf
orient(fig2,'landscape')
print(name1,'-fillpage','-dpdf');

%store everything in an excel file
averagefps=mean(fps(~isnan(fps))); %exclude the NaN samples
maxrow=max([length(STD{1}),length(STD{2}),length(STD{3}),length(STD{4}),length(STD{5
})]); %take the longest
for k=1:5
    STD{k}(length(STD{k}):maxrow,1)=NaN; %make everything equally long => NaN will
be set to empty in excel
end

%make a table and then write it in an excel file
T=table((1:maxrow)'/(averagefps*60),round((1:maxrow)'/
(averagefps*60)),STD{1},STD{2},STD{3},STD{4},STD{5},...
```

```
    'VariableNames',
{'Minutes','RoundedMinutes','LampSTD','STD1','STD2','STD3','STD4'});

filename = strcat(name1,'.xlsx');

writetable(T,filename,'Sheet',1)



%go one level up in the directory

%cd ..
```

Scritp 3: RunMe.m

```
%% prepare

clear all;

close all;

addpath('.');

addpath('mmread');


%% The directory that we are working on - we use name1 since name is in the .mat
files

%name1='HB005_2018-05-02';

%name1='HB016_2018-05-10';

%name1='HB128_2018-05-30';

%name1='HB137_2018-05-04';

%name1='HB148_2018-05-01';

%name1='HB163_2018-05-17';

%name1='HB163_2018-05-22';

%name1='HB175_2018-06-04';

%name1='HB272_2018-06-05';

%name1='HB272_2018-06-11';

%name1='HB289_2018-05-28';

%name1='HB289_2018-05-31';

%name1='HB293_2018-05-29';

%name1='HB296_2018-05-18';
```

```
%name1='HB302_2018-05-14';

%name1='HB303_2018-06-08';

%name1='HB322_2018-06-08';

%name1='HB343_2018-06-01';

%name1='HB352_2018-05-24';

%name1='HB352_2018-05-28';

%name1='HB352_2018-06-01';

%name1='HB354_2018-05-08';

%name1='HB354_2018-05-16';

%name1='HB358_2018-05-06';

%name1='HB374_2018-05-15';

%name1='HB409_2018-05-10';

%name1='HB409_2018-05-16';

%name1='HB739_2018-05-03';

%name1='P002_2018-05-18';

%name1='P003_2018-05-08';

%name1='P003_2018-05-20';

%name1='P008_2018-05-20';

%name1='P008_2018-05-24';

%name1='P010_2018-05-14';

%name1='P010_2018-05-23';

%name1='P011_2018-05-01';
```

```
%name1='P014_2018-05-22';

%name1='P014_2018-05-26';

%name1='P015_2018-06-11';

name1='U001_2018-05-03';



%% open the connection

f = ftp('ieeservftp01.unibe.ch','gschuster','hah@J!iay7vu');


%% get that directory

mget(f,name1)


%% use the same code as before

cd(name1)


temp=strcat('Lamp_',name1);

if exist(strcat(temp,'.mp4'),'file')

   disp(temp)

   calculateandstore2(temp);

end


prename='PosX_';
```

```
for k=1:4

    prename(4)=num2str(k);

    temp=strcat(prename,name1);

    if exist(strcat(temp,'.mp4'),'file')

       disp(temp)

       calculateandstore2(temp);

    end

end


%% call the display script

displayResults2


%% move the two pdfs, the mat file and the excel file to the ftp server

cd(f,name1);

mput(f,'*.mat');

mput(f,'*.pdf');

mput(f,'*.xlsx');

cd(f,'..');

close(f);


%clean up the local copies

delete('*.*');
```

```
cd('..');

rmdir(name1);
```

Source of the mmread function:

https://ch.mathworks.com/matlabcentral/fileexchange/8028-mmread
